# Supplementary figures and images for: Highly Constrained Kinetic Models for Single-Cell Gene Expression Analysis
Source: bioRxiv. 2026 May 29:2026.05.22.727214. Preprint. [Version 2] doi: 10.64898/2026.05.22.727214 (PMC13232213; doi:10.64898/2026.05.22.727214)

Figure S1.

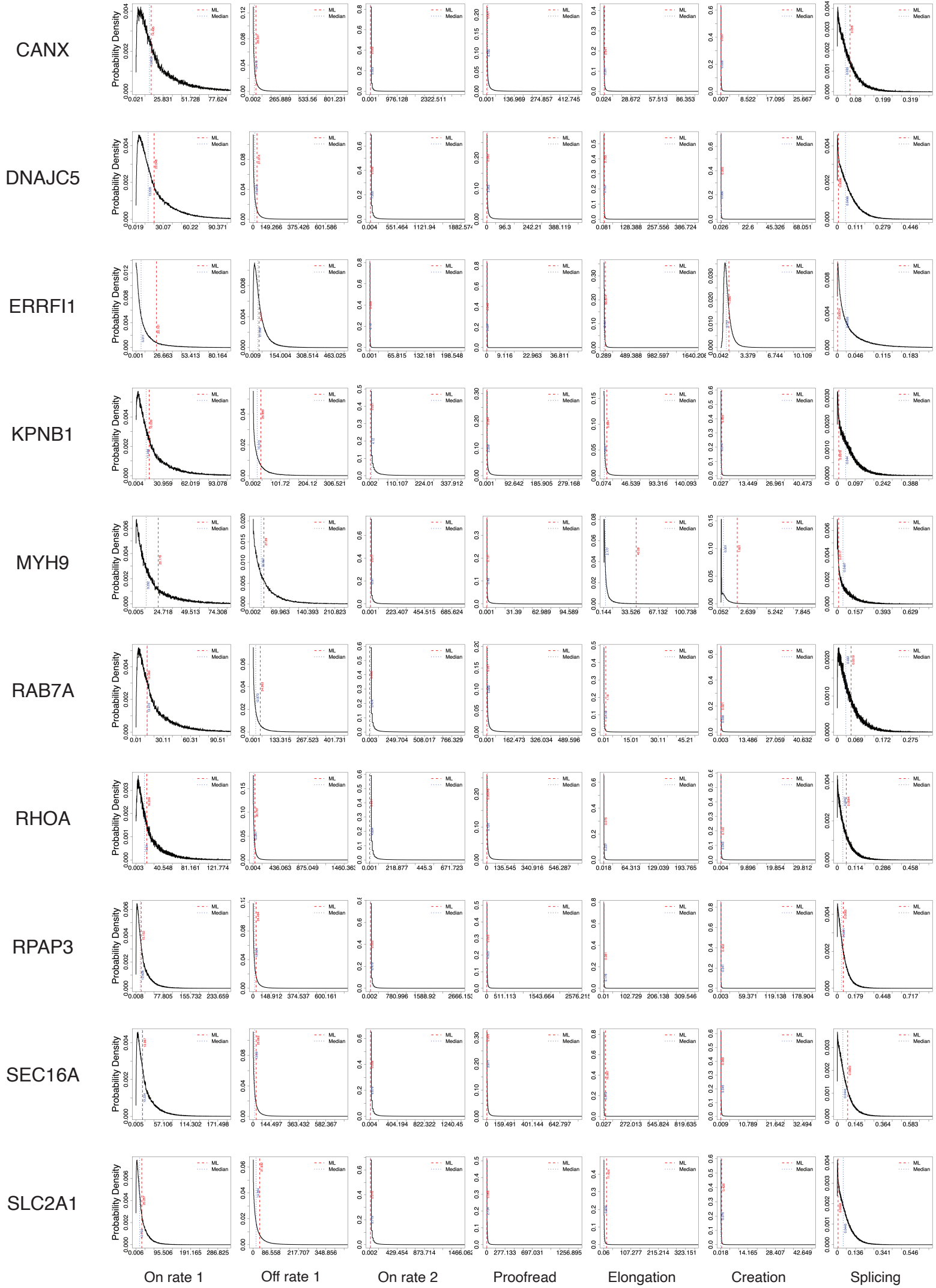

Figure S2.

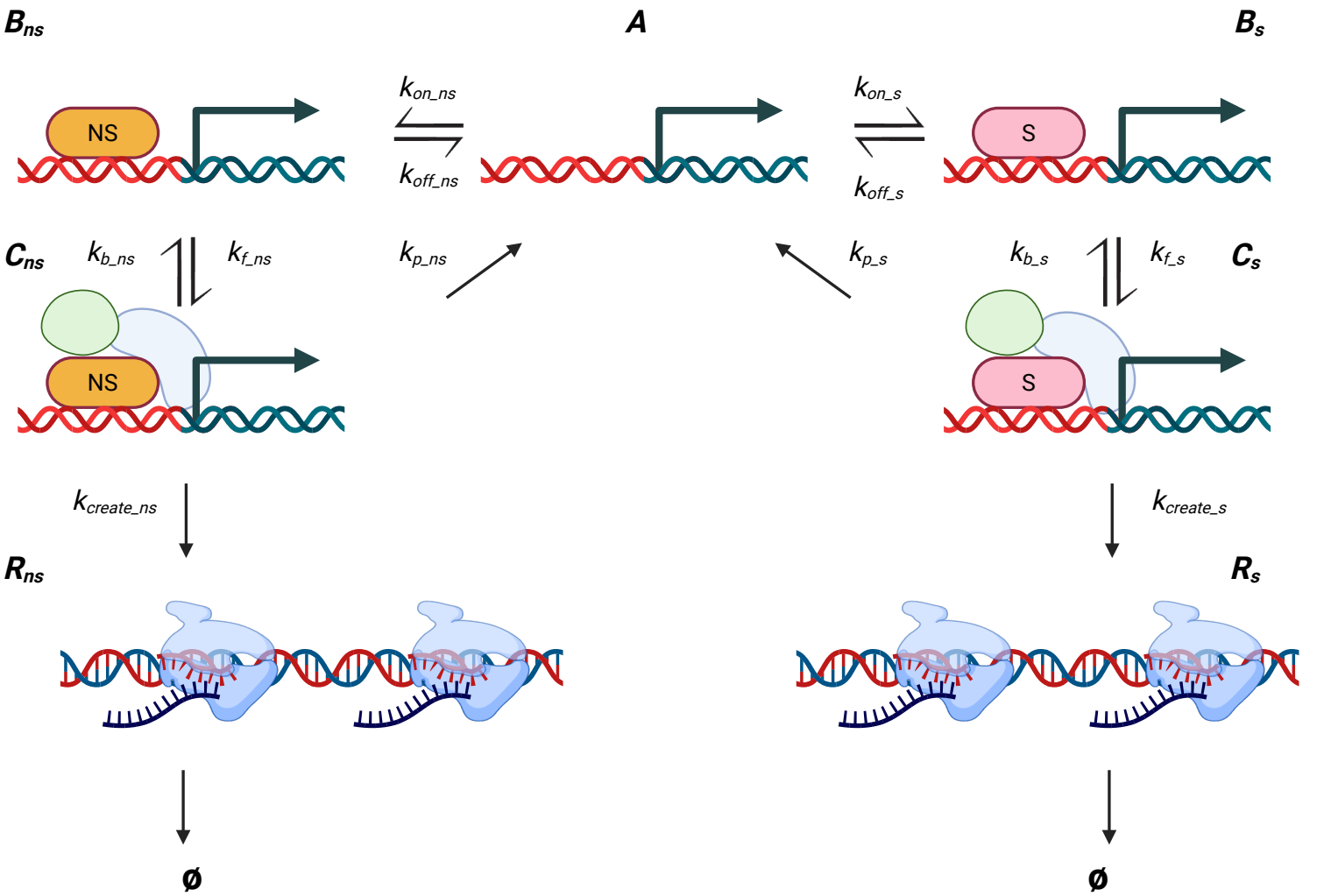

Supplement: Supplement 1 — Figure S1. Posterior distributions of the fitted rates Posterior distributions of the fitted rates for the 10 genes: CANX, DNAJC5, ERRFI1, KPNB1, MYH9, RAB7A, RHOA, RPAP3, SEC16A, and SLC2A1. Red dashed line represents the maximum likelihood of the fitted rates, and blue dotted line indicates the median of the fitted rates. Figure S2. Comparison between specific and non-specific TF binding in the KP model Illustration of the KP model with non-specific (ns) and specific (s) binding of TFs. [file media-1.pdf]
